# “An Incomplete Theory of the Mind”

**DOI:** 10.3389/fpsyg.2012.00418

**Published:** 2012-10-19

**Authors:** Javier Bernacer, Jose Ignacio Murillo

**Affiliations:** ^1^Mind-Brain Group, Institute for Culture and Society, Universidad de NavarraPamplona, Spain

Clark offers a detailed description of certain current views of the brain that define it as a “prediction machine.” According to these accounts, it is a hierarchically organized processing unit whose main role is elaborate top-down predictions about sensory inputs, and compute a bottom-up prediction error (PE) which will help refine future predictions. The ultimate stable state, says Clark citing Mumford (1992), would be the delivery of a signal from the cerebral cortex to lower areas which would fully predict the sensory information they are receiving. In this state, the PE signal would not exist at all. In Clark’s opinion, the brain is a “black box” which receives information from the world without a direct access to it: “*[the brain] must discover information about the likely causes of impinging signals without any form of direct access to their source*”; that is, it just perceives the perturbations that these signals are causing on its own state, makes predictions about the nature of the signals, and elaborates a response.

In our opinion, the main problem of this view is the description of cognition as prediction, and prediction as some sort of representation. Perception, hence, is just a mean to correct previous predictions. This eludes the question about the very nature of cognition. In fact, if we understand cognition as a representative activity, there are two possible interpretations: cognition could be either (1) the process that generates the representation or (2) the resulting representation itself. In (1) we identify cognition with a causal process, and this neglects the fact that a representative activity is only possible if guided by a previous cognition. In (2) we face the problem of explaining what is *to know* the representation: if cognition is a representation, we need a representation to know the previous one, and so on *in infinitum*. However, inasmuch as cognition goes beyond the mere generation of a prediction, a representation can be compared with the reality that represents. This comparison is a cognitive activity irreducible to the representation itself.

In addition, *representation* is not possible without some kind of previous “presentation.” Clark’s example of the black box is very illustrative. He overlooks that cognition itself means the suppression of the “black box problem.” The confinement of an entity into a “black box” is a problem only if that entity is a cognitive system; that is, only a unit with the ability of cognition can be deprived of the possibility of comparing a representation with the external reality that represents. We think this clarification is especially important to understand the more basic sensory and perceptual levels. Vision, for instance, is not a representation of the world, but a codification of some information *about* the world. This intrinsic relation between cognition and the world can be called *intentionality*, using a word reintroduced by phenomenology, but only if, unlike phenomenology (Gallagher and Zahavi, 2008), we disentangle intentionality from consciousness. Following the example, vision does not need to be conscious to be cognition; it just need to apprehend some information (i.e., color) that is in relation to the world. This could be considered a “presentation” (in a weak sense) of reality.

This understanding of the cognitive activity is necessary to accept the existence of representations, which are an important way of knowledge, although they are not the first level. Assuming this, the interaction between the brain and the world has not only an effective or impinging character, as Clark states. The basic and more general level of cognition consists of the abstraction or separation of “information” by sensory systems from the effective causality which transports it (Polo, 2002). This process generates information as such, and makes it available to superior levels of cognition and to behavior. In our opinion, such aspect of cognition is not explained by the Bayesian modeling of the brain.

One of the consequences of this omission is that the unified science of mind and action Clark proposes makes a deficient distinction between cognition and action. Action is a causal intervention of the living being in the world, but it is only an action inasmuch as it is informed by cognition. This leads to a different, although connected, sense of intentionality (Moya, 1990). The dissolution of cognition in a causal effective process blurs, as a consequence, the distinctive features of actions.

However, the representational model proposed by Clark could be useful to understand some sort of secondary levels of cognition and practical action. In fact, we would like to propose a neural substrate for the so-called “Bayesian brain.” This should be a brain region able to integrate the top-down prediction with the bottom-up PE. Clark cautiously proposes a possible candidate for such interaction between predictions and PEs citing Friston (2005, 2009) and Mumford (1992) models. However, we believe a different option should be taken into account.

The striatum is the main receptor of cognitive, sensory, motor, and emotional information within the basal ganglia, a group of nuclei intimately involved in action from a wide point of view (decision-making, action selection, procedural memory, and instrumental learning, among others; Redgrave and Gurney, 2006). This special involvement in action puts the striatum in a preferential position to be a biological substrate of the “Bayesian brain.” On the other hand, midbrain dopaminergic neurons located in the substantia nigra pars compacta and ventral tegmental area have been reported to convey a PE signal to striatal areas (Schultz, 1998). Its representation in the striatum has been widely demonstrated in humans (Tobler et al., 2006). In fact, Clark mentions the role of this nigrostriatal PE signal in different sections of his article. Moreover, anatomical studies have also demonstrated that the same striatal neurons targeted by a dopaminergic input also receive projections in the same synaptic spine from associative, motor, and limbic cortices – those possibly carrying the predictions Clark mentions in his model (Gerfen and Surmeier, 2011). The result of this interaction between the neural signals of priors (from the cortex) and errors (from the midbrain), together with the modulation from striatal interneurons (Bernacer et al., 2012), could determine the activity of striatal projection neurons, thus facilitating or inhibiting the performance of an action.

In conclusion, a holistic theory of the mind should distinguish cognition from action in a sharper way than Clark does. Action is a causal process arising in the subject and aimed to the outside world, but it is also guided by cognition itself. This is the key feature of action. We also believe there are enough evidences to propose the striatum, part of the basal ganglia, as the neural substrate of Clark’s proposal on practical action. On the other hand, the difficulty of disentangling information from causality in laboratory experiments makes difficult to find a neural substrate for sensory cognition.
